# Ring chromosome 18 abnormality in acute myelogenous leukemia: the clinical dilemma

**DOI:** 10.1186/1756-8722-3-25

**Published:** 2010-07-22

**Authors:** Shanthi Sivendran, Stephen Gruenstein, Adriana K Malone, Vesna Najfeld

**Affiliations:** 1 Division of Hematology/Oncology, Departments of Medicine, The Tisch Cancer Institute, The Mount Sinai School of Medicine, New York, NY, USA; 2 Department of Pathology, The Mount Sinai School of Medicine, New York, NY, USA

## Abstract

The ring chromosome is a circular, structural abnormality composed of either multiple chromosomes or a single chromosome with loss of genetic material at one or both ends. This chromosomal rearrangement is often unstable with frequent recombinations and may be accompanied by either loss or amplification of genetic material[1]. Considering that ring chromosomes are rare in acute myelogenous leukemia (AML), it is difficult to risk stratify patient prognosis, particularly when the ring chromosome occurs as the sole abnormality. Here we report a case of a ring chromosome 18 abnormality in a patient with newly diagnosed AML with monocytic differentiation. Cytogenetic analysis demonstrated 46, XY, r(18)(p11q21) karyotype in 19 of 34 evaluated metaphase cells. The patient received induction chemotherapy and subsequent allogeneic cord blood transplant from a sex-matched donor, and remained in hematologic and cytogenetic remission for 120 days post transplant. Soon after, he developed post transplant lymphoproliferative disorder and died of multi-organ failure. Although r(18) chromosomal abnormalities were not classified in the recent updated evidence-and expert opinion-based recommendations for the diagnosis and management of AML (likely due to the small number of reported cases), the patient was treated as high risk with stem cell transplantation. This was based on the unstable nature of the ring chromosome and the poor outcomes described in the literature of patients with sole ring 18 abnormalities.

## Background

Prognostic features in AML are strongly influenced by genetic changes in leukemic cells[2]. Currently, based on cytogenetic findings and mutational status of some genes, patients are stratified into favorable, intermediate, and unfavorable risk categories[2]. These categories are key determinants for attainment of complete remission and overall survival[2]. Recent updated evidence-and expert opinion-based recommendations for the diagnosis and management of AML have provided further categorizations of AML based on pretreatment chromosomal abnormalities[3]. However, there are rare, recurrent, cytogenetic abnormalities in AML that have not been classified. This is primarily due to the small number of reported patients, whose risk category and response to treatment is not well known. Ring chromosomes are rare cytogenetic abnormalities that occur in less than 10% of hematopoietic malignancies but have been reported in up to 70% of mesenchymal tumors[1]. They vary in size, shape, and number. Only two patients were reported with AML and a ring chromosome 18 abnormality[4,5]. In this report we describe a patient with M5 AML with a ring 18 abnormality and discuss the etiology, clinical features, classification, and the clinical dilemma related to treatment of ring chromosome aberrations in AML.

## Case presentation

A 36 year old man presented with a one month history of nausea, loss of appetite, diarrhea, night sweats, and a twelve pound weight loss. He had no significant past medical history and, despite his work in construction, denied any previous chemical or radiation exposure. Peripheral blood revealed anemia (Hb 8.2 g/dL), thrombocytopenia (38 × 10^3^/uL) and a white cell count of 2.6 × 10^3^/uL. A bone marrow biopsy demonstrated a markedly hypercellular marrow (90-100% cellularity) with increased mature and immature granulocytes and atypical megakaryoctyes. The bone marrow aspirate contained myeloblasts, monoblasts, and promonocytes accounting for 36% of the cellularity. Significant dysplasia was present in the myeloid and erythroid lineages. Flow cytometry demonstrated an abnormal monocytic population characterized by HLA-DR+, CD13 partial+, CD64+, CD4dim+, and negative for CD14, suggestive of immaturity and reported to be a feature of abnormal monocytes in AML[6]. Most CD 117 + myeloblasts and/or monoblasts were negative for CD34, another marker of immature cells which is usually negative in AML with monocytic differentiation[6]. The diagnosis was consistent with acute monocytic leukemia.

**Figure 1:**
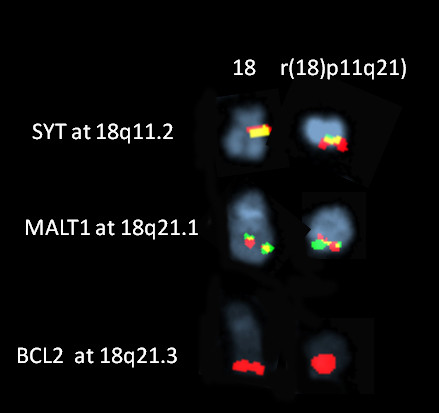
**Metaphase FISH maping of *SYT*, *MALT1 *and *BCL2 *on normal chromosome 18 (left)**. *SYT *and *MALT1 *FISH probes were "breakapart" dual color probes where the 3' end of the probe and the 5' end of the probe were labeled in two different colors (red and green) while the BCL2 was a locus specific probe labeled in red. The mapping of the loci on ring (18) (right) revealed all three loci intact with apparent no loss of genetic material from these loci on the ring 18 chromosome.

Cytogenetic analysis demonstrated 46, XY, r(18)(p11.1q22) karyotype in 19 of 34 evaluated metaphase cells. Interphase fluorescence in situ hybridization (FISH) evaluation revealed *BCR-ABL1, PML-RARA, RUNX1-RUNXT1 *fusion negative in all cells. *CBFB *and *MLL *(Probe manufacturer: Abbott Molecular Diagnostics - Des Plaines, Illinois) rearrangements were not detected. These are recurrent genetic changes associated with AML and included in our AML panel for prognostic purposes. Metaphase FISH studies demonstrated that *BCL2*, S*YT*, and *MALT1 *were present and localized to the long arm of chromosome 18 and were not lost during the ring formation as shown in Figure 1. Additionally, the patient was found to be FLT3 negative and NPM1 positive.

The patient received induction chemotherapy on CALGB protocol 10503 with cytarabine arabinoside 100 mg/m2/day, daunorubicin 90 mg/m2/day, and etoposide 100 mg/m2/day. A repeat bone marrow aspirate and biopsy was consistent with a hypocellular marrow with no evidence of disease. Four cytogenetic analyses following induction chemotherapy demonstrated a normal 46, XY karyotype. The patient underwent a conditioning regimen of melphalan, thiotepa, fludarabine, and ATG followed by an allogeneic cord blood transplant from a sex matched donor. He remained in hematologic and cytogenetic remission with 100% donor cell engraftment for 120 days. Soon afterwards he developed post transplant lymphoproliferative disorder and died of multi-organ failure.

## Discussion and Conclusions

In patients with hematopoietic malignancies, ring chromosomes are commonly part of a complex karyotype. Only two patients with AML and an isolated ring 18 abnormality have been reported in the literature. The first case, a 47 year old male patient with AML-M4, had cytogenetic analysis performed on bone marrow cells after induction chemotherapy with 46, XY, r(18)(p11q23) karyotype and 9% of these cells contained a r(18). Breakpoints were not documented. This patient had greater than eighteen months of survival following diagnosis[4]. The second patient had extensive chemotherapy and radiation for Hodgkin lymphoma and developed therapy related AML with 46, XX, r(18)(p11q21) karyotype. His response to chemotherapy was poor[5]. It is important to note that both of these cases are over 20 years old and there is lack of documentation on these patients since the original reports. Additionally, given the advancements in cytogenetic analyses since these original reports, there was a chance for chromosome misidentification at the time.

Ring chromosomes occur when the two ends of a chromosome fuse together and form a ring shape. There are several ways in which this can occur. Breaks in the chromosome arms and fusion of the proximal broken ends can lead to ring formation with loss of distal chromosomal material. The cause of these DNA breaks and ligation of the ends is unknown. Alternatively, rings can be formed by telomere dysfunction. This occurs when the terminal ends of a chromosome fuse without significant loss of genetic material. Animal models and in vitro studies have shown that the mechanism of telomeric ring formation may be secondary to detachment of protective proteins on the chromosome ends when shortening of telomeric DNA occurs[1]. The above two scenarios produce rings that do not necessarily produce amplified sequences. Rings that produce amplified sequences can also occur. This happens through the "break-fusion-bridge" which leads to frequent recombination events[1].

Pretreatment cytogenetic analysis constitutes an independent prognostic determinant for response to treatment, risk of relapse and overall survival in AML[2]. It is a crucial factor in selecting further therapy. In patients under the age of 60 with favorable cytogenetics, successful induction chemotherapy followed by maintenance therapy will produce a durable remission in up to 60% of patients[7-9]. This is in stark contrast to patients with unfavorable cytogenetics who have a durable remission of 12% given the same therapy[7-9]. These patients are recommended to undergo either a matched sibling transplant, alternative donor hematopoietic stem cell transplant, or receive treatment on a clinical trial[7-9]. Given the paucity of information for patients with a ring chromosome, it is difficult to risk stratify and classify these patients according to the recently updated international expert panel guidelines[3]. Of the ten reported cases of ring 18 abnormalities in acute myeloid leukemia, few survived[1,4,5]. Eight of these patients had multiple, complex cytogenetic abnormalities which are associated with a poor prognosis. One of the cases of isolated r(18) discussed above had therapy related AML, which in multivariate analysis remains an adverse risk group. A summary of the karyotypes, treatments, and follow-up is presented in Table 1. One could speculate that due to the instability of the ring chromosome and the potential for multiple chromosomal rearrangements, including deletions or amplifications, these patients could be classified in an intermediate or unfavorable risk category. Three significant trials have examined the association of cytogenetic analysis and survival outcomes in AML. Of these, no specific mention is made of ring chromosome aberrations. Both the Southwest Oncology Group/Eastern Cooperative Oncology group and Cancer and Leukemia Group B studies categorize all cytogenetic abnormalities that are rare in an "unknown" category and a risk assessment for these abnormalities was not determined[7,8]. The Medical Research Council AML 10 trial groups all patients with "structural" abnormalities into one category and based on complete response, survival, and relapse rates determined these abnormalities were of intermediate risk[9].

**Table 1:** Literature review of ring 18 cases in acute myelogenous leukemia

Karyotype [Number of cells]	Antecedent disease	Disease	Treatment	Followup	Reference
45, XY, der(1)t(1;11)(p36;q23), -11, dmin(18)46, XY, r(18), dmin(1) [NA]/46, WY, dmin(1)	MDS	AML-M4	ARA-C	Died	Michalova et al[10]
46, XX, r18(p11q21) [NA]/46, XX	Hodgkin's disease	NA	NA	NA	Lee et al[4]
46, XY, r(18)(p11q23)[9%]	NA	AML-M4	NA	18+months	Testa et al[5]
43-46, XX, -3, -5, -7, +8, r(18), +20, dmin[cp19]/46, XX[1]	Multiple myeloma	AML	VAD MP VMCP VBAP	NA	Sawyer et al[11]
r(18) and r(20) present. Full karyotype not available	NA	AML	NA	NA	Gisselsson et al[12]
47, +21, r(?18)[31]	NA	AML-either M1 or M2	NA	NA	Fitzgerald et al[13]
39-44, Y, der(X)t(X;9) (p11.2;?q32), del(1)(p13p32), t(1;10)(p21;q26), der(5)t(X;5)(p11.2;q11), -9, der(?16)r(16; 18)(?;?q11?q12), der(17)t(9;17)(?;p11), -18[cp3]	NA	tAML	NA	NA	Mrozek et al[14]
44, XX, del(2p), del(5q), - 7, - 9, - 14, der(l7]<i(l7p)?>, -18, r(18), - 21, + 22, + mar.variations [21]	NA	AML-M4	NA	NA	GFCH[15]
Ring 18 present but karyotype not available so unclear if this is a sole abnormality	NA	NA	NA	NA	Schoch et al[16]
45, XX, -7. r(18)[15]	NA	AML-M6	NA	Lost to followup	Gibbons et al[17]

NA: not available; VAD: vincristine, adriamycin, dexamethasone; MP: melphalan, prednisone; VMCP: vincristine, melphalan, cyclophosphamide, prednisone; VBAP: vincrisitne, carmustine, doxorubicine, prednisone; CY: cyclophosphamide; TBI-F10: total body irradiation in fractionated doses of 10 Gy

Our patient had ring 18 as a sole abnormality without the loss of *SYT, MALT1 *and *BCL2 *by metaphase FISH. This is important because *MALT1 *and *BCL2 *are genes that regulate cell proliferation. To our knowledge, this case is the first ring 18 abnormality demonstrating that key genes were not lost during the ring formation. Although it is not possible to risk stratify this patient based on current guidelines, the patient was treated as high risk with stem cell transplantation. This decision is based on the unstable nature of the ring chromosome, previous trials conducted by the Medical Research Council, and the poor outcomes described in the literature of patients with sole ring 18 abnormalities. This case contributes to a growing body of literature to help risk stratify patients and guide treatment options in patients with ring abnormalities and acute myelogenous leukemia.

## Consent

Written informed consent was obtained from the patient's next of kin for publication of this case report and any accompanying images. A copy of the written consent is available for review by the Editor-in-Chief of this journal.

## Competing interests

The authors declare that they have no competing interests.

## Authors' contributions

SS was responsible for manuscript preparation and was involved in patient care. SG and AG were responsible for patient care and manuscript review. VN performed cytogenetic analysis and was involved in manuscript preparation. All authors have read and approved the final manuscript.
